# A microbial inhibition assay in microplates using *Bacillus licheniformis* for detection of enrofloxacin and sulfamethazine in chicken spiked kidney, liver and muscle tissues

**DOI:** 10.1002/vms3.1293

**Published:** 2023-10-04

**Authors:** Sara Mirzaie, Fahimeh Jamiri, Majid Javanmard Dakheli, Saeed Mirdamadi

**Affiliations:** ^1^ Department of Agriculture Iranian Research Organization for Science and Technology (IROST) Tehran Iran; ^2^ Department of Pathobiology Faculty of Veterinary Medicine University of Tehran Tehran Iran; ^3^ Department of Chemical Technologies Iranian Research Organization for Science and Technology (IROST) Tehran Iran; ^4^ Department of Biotechnologies Iranian Research Organization for Science and Technology (IROST) Tehran Iran

**Keywords:** antibiotic, detection limits, microbial inhibition assay, MRL, poultry products

## Abstract

**Background:**

The presence of antimicrobial drugs residues in animal products at levels higher than the maximum residue level (MRL) may have adverse effects on consumer health such as allergic reactions and resistance development. Therefore, it is necessary to monitor animal products for the presence of antimicrobial residues.

**Objectives:**

The aim of this study was to evaluate the detection limit of microbial inhibition assay (MIA) in microplate by using of *Bacillus licheniformis* as indicator microorganism for two antibiotics, enrofloxacin (ENR) and sulfamethazine (SMT), in broiler chicken's kidney, liver and muscle tissue samples.

**Methods:**

Spiked tissues samples for the two antibiotics were analysed separately by this method. The results of the assay were evaluated by the determination of the absorbance after mean 3.47 h of incubation at 45°C.

**Results:**

Results showed that the detection limits of MIA for ENR and SMT in kidney (124.03 and 23.21 μg/kg, respectively) and liver (90.02 and 62.03 μg/kg, respectively) as well as SMT in muscle (46.95 μg/kg) were lower than EU (European Union) – MRL, whereas the detection limit for ENR in muscle was slightly higher than MRL (136.3 μg/kg compared to 100 μg/kg MRL). Furthermore, the MIA in the current study was found to be more sensitive to SMT than ENR (92% and 88% sensitivity rate, respectively). No false‐positive was observed in the assay.

**Conclusions:**

Based on the results, the MIA investigated in this study had the potential to detect ENR and SMT residues in broiler chicken kidney, liver and muscle tissues at levels below or close to EU – MRL but offered lower capability for the detection of ENR compared with SMT in kidney and muscle tissue samples.

## Graphical abstract

Microbial inhibition assay (MIA) using *Bacillus licheniformis* detected ENR and SMT in chicken spiked tissues at lower levels than MRLs. The MIA was more sensitive to SMT than ENR, whereas the specificity rate was 100%. The assay offered lower capability for ENR compared with SMT detection in chicken kidney and muscle tissues.

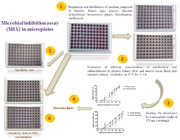

## 1 INTRODUCTION

Antimicrobial drugs are widely used in poultry as well as other food‐producing animal productions for therapeutic and prophylactic purposes against infectious diseases. It has been reported that global consumption of antibiotics in animals is twice that of humans (Van Boeckel et al., 2015). Furthermore, it has also been predicted that the application of antimicrobials for livestock and poultry throughout the world will increase 67% from 6315 tons in 2010 to 105,596 tons by 2030 (Falowo & Akimoladun, 2019). Many antimicrobial drugs, including sulfonamides, tetracyclines, quinolones, beta‐lactams, aminoglycosides and macrolides, are warranted to be used in food animals from which sulfonamides and quinolones are among the most commonly prescribed antimicrobials for commercial poultry in Iran (Baniasadi et al., 2019; Madadi et al., 2014).

Non‐prudent use of antibiotics in animal and poultry production such as using large doses, consistent use and ignoring withdrawal period could result in deposition of drug residues in different tissues of animal which threaten consumer's health (Sanz et al., 2015). The presence of chemotherapeutic drug residues in animal‐derived food may cause various health problems including hypersensitivity reactions, interference in the normal gut flora and development of resistant bacteria for the consumers (Ezenduka et al., 2014). Therefore, monitoring of veterinary drug residues in foods of animal origin is important to prevent potential hazard of residues to public health. To prevent the risk of antimicrobial residues in animal products, maximum residue limit (MRL) which is the maximum permissible level of residues in the food with no adverse effect on consumer health was set by international organizations and associations such as WHO, FAO and European Community (Lateefat et at., 2022).

Several tests, including microbiological, chromatography‐based, immunological, physicochemical and biological sensing systems, have been developed for the detection of antimicrobial drug residues in animal products (Chen et al., 2017; Gondová et al., 2014; Ramatla et al., 2017). Among them, microbial assays are used largely for monitoring of antimicrobial drug residues because these methods are inexpensive and have detection limits close to the MRL for beta‐lactams, fluoroquinolones, tetracyclines and sulfonamides. Microbial inhibition assays (MIA) can be divided into two test tube and petri dish methods. Test tube‐based assays provide a colorimetric response with visual interpretation or absorbance measurement in a short incubation time (3–4 h) and are more applicable for screening of antimicrobial residues in large number of samples compared with petri dish methods relying on determination of inhibition zone after 16–18 h of incubation (Tumini et al., 2019).

Using of antimicrobial residues screening tests will reduce the number of samples needed to be evaluated by a more sensitive confirmatory test (Ezenduka et al., 2014). It is important that animal products be assessed by a method with good detection capability at or below MRL for most of the antimicrobial drugs used in food animal production. Several commercial kits based on MIA in test tubes have been developed for screening animal products. These tests include Brilliant black reduction test (Molina et al., 2003), PremiTest (Stead et al., 2004), Copan milk test (Le Breton et al., 2007), Delvotest (Althaus et al., 2003), Eclipse 100 (Beltrán et al., 2015) and Charm Blue‐Yellow II (Linage et al., 2007). However, no ideal microbiological test exists for the detection of all types of common antimicrobials used in various animal products. Most of these current microbial screening tests were developed to detect antimicrobial residues in milk and are based on the inhibition of *Geobacillus stearothermophilus* as an indicator microorganism because it grows in a short time (Beltrán et al., 2015; Wu et al., 2019). However, it is not sensitive enough to many commonly used antibiotics except beta‐lactams (Nagel et al., 2013). Hence, in some studies methods made up of other microorganisms such as *Bacillus megaterium*, *Bacillus cereus*, *Bacillus subtilis* and *Bacillus licheniformis* that are sensitive to different groups of antimicrobials have also been proposed. It has been reported that *B. licheniformis* ATCC 14,580 has acceptable sensitivity to both quinolones and sulfonamides (Nagel et al., 2013; Tumini et al., 2019).

According to European legislation, MRL for sulfonamides in muscle and other organs of poultry is 100 μg/kg, and MRLs for quinolones in kidney, liver and muscle of poultry are 300, 200 and 100 μg/kg, respectively. By using of a microbiological inhibition test on petri dishes (four‐plate test), it has been reported that the prevalence of antimicrobial residues in poultry tissues in Iran was 39.41% (Mohammadzadeh et al., 2022). By analysing of chicken meat samples, Dabagh Moghadam et al. (2017) revealed that 100% and 80% of samples were contaminated with fluoroquinolone and sulfonamides respectively and 11.43% of samples exceeded the MRL.

To the best of our knowledge, there was no report regarding development of a microbiological system in microplates using *B. licheniformis* as indicator microorganism for the screening of poultry samples for the presence of antimicrobial drug residues. As indicated earlier, previous studies pointed out that *B. licheniformis* is sensitive to quinolones and sulfonamides and may detect these antimicrobials at lower levels compared with *G. stearothermophilus*. Therefore, the goal of the present study was to assess MIA with *B. licheniformis* as an indicator microorganism in microplates for rapid detection of residues of enrofloxacin (ENR) and sulfamethazine (SMT) in chicken tissues.

## 2 MATERIALS AND METHODS

### 2.1 Antimicrobials

Standard antimicrobial agents, ENR and SMT were obtained from Sigma Chemical Co. Stock aqueous solutions were prepared in sterilized distilled water at a concentration of 1 mg/mL by using of NaOH 0.1 mol/L as a solvent and stored at  −20°C until use. Working solutions were prepared by applying suitable dilutions of the stock solutions at a concentration of 5 μg/mL at the time of analysis to prevent possible alteration of antimicrobial properties.

### 2.2 Preparation of indicator microorganism and MIA microplates

*B. licheniformis* ATCC 14580 (PTCC 1721 strain) was provided from Persian type culture collection centre, Iranian Research Organization for Science and Technology (IROST), Tehran, Iran. The organism was cultured in nutrient agar with 10 mg/L MnCl_2_, 100 mg/L CaCl_2_ and 500 mg/L MnSo_4_. H_2_O incubated in 45°C for 72 h. At the end of incubation period, endospore formation was assessed by Schaeffer–Fulton staining method. The bacterial cells were washed and collected from the medium surface by using of a sterile cell scraper and sterilized distilled water. Harvested cell suspension was washed three times in sterilized distilled water by centrifuging at 4000 rpm for 15 min at 4°C and was stored in refrigerator (4°C).

To prepare MIA microplates, Mueller–Hinton agar (Merck) was used. The culture medium was sterilized at 121°C for 15 min. After the medium was cooled down to 50°C, glucose (10 g/L), *B. licheniformis* spore suspension (1.18 × 10^8^ spores/mL), bromocresol purple (50 mg/L) and sensitivity improvers (80 μg/L trimethoprim and 600 μg/L ENR) were added. After the medium ingredients were mixed well, a 100 μL of medium was dispensed into each well of 96 wells microplates by using of a multi‐channel pipette and then the plates were sealed with aluminium foil and stored at 4°C until use (Nagel et al., 2009).

### 2.3 Blank chicken cases, preparation of tissue fluids and spiked samples

The chicken sampling was carried out according to the ethical guidelines for animal use approved by the Animal Care Committee of the Iranian Council of Animal Care 1995 (No. IACUC95). During this study, a total number of 50 6‐week‐old Ross 308 broilers with a history of no antimicrobial drug used during rearing period were provided from chicken farms of Animal Science Research Institute of IRAN (ASRI) and University of Tehran (UT). Samples, including whole kidneys, livers and parts of breast muscle tissues, were collected from slaughtered birds by using of sterile scissors and forceps, packaged in separate plastic bags and transported in a coolbox to the laboratory where they kept frozen at −20°C until use. To prepare tissue fluids, samples were homogenized with distilled water at a ratio of 1:2 (tissue:distilled water) and incubated in water bath for 10 min at 80°C to impair natural bacteriostatic substances in chicken tissues. The mixture was then centrifuged at 5000*g* for 10 min. The resulting supernatant was transferred to a new clean tube and kept frozen at −20°C until the analysis (Wachira et al., 2011; Mwangi et al., 2011). Spiked samples, including antimicrobials diluent (sterilized distilled water) as standard solution and homogeneous kidney, liver and muscle tissue fluids, were prepared by adding antimicrobial working solutions to antimicrobial‐free sample fluids at the spiked levels shown in Table 1.

**TABLE 1:** Spiked levels of sulfamethazine and enrofloxacin employed in different matrixes.

Antimicrobial agents	Matrixes^a^ (tissue fluids)	Spiked levels (μg/kg)
Sulfamethazine (SMT)	All tissues	5, 10, 50, 100, 200, 400, 800, 1000
	Kidney	5, 25, 150, 300, 900, 1800, 3000, 4000
Enrofloxacin (ENR)	Liver	5, 25, 75, 200, 400, 800, 2000, 3000
	Muscle	5, 10, 50, 100, 300, 900, 2000, 3000

^a^ Different spiked levels were also prepared in distilled water as antimicrobial agent diluent (standard solution) in addition to tissue fluids.

### 2.4 Determination of the detection limits

**FIGURE 1:**
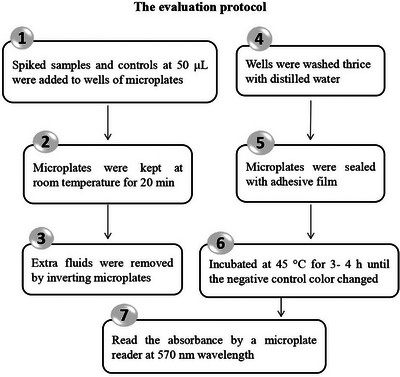
Flow chart illustrating the evaluation protocol of microbial inhibition assay in microplate.

To assess the detection limits of MIA in microplates, the spiked samples in 11 replicates of 8 concentrations were evaluated so that negative result in the first 2 concentrations and positive results in the last 3 concentrations could be observed. Antimicrobial‐free tissue fluids were considered the negative control, and tissue fluids samples with 100 μg/mL ENR and 400 μg/mL SMT were taken for the positive control. Positive and negative controls were tested in four replicates. The evaluation protocol was illustrated briefly in Figure 1. A 50 μL of spiked samples and controls were added to respective wells of microplates. The plates were kept at room temperature for 20 min to allow samples to diffuse into the medium. The remaining fluid on the medium surface was removed by inverting microplates on sterilized tissue papers, and the wells were washed three times with distilled water. Finally, the microplates were sealed with adhesive film and incubated at 45°C for 3–4 h until the negative control colour turned pale and yellow (Wu et al., 2019). At the end of the incubation time, the absorbance was measured by using of a microplate reader (Biotek cytation 5) at 570 nm. Samples were considered ‘positive’ when they remained degrees of purple colour and ‘negative’ when the colour faded towards yellow. A colour change from purple to yellow indicated that the sample contained no antimicrobial residues or that the concentration of antimicrobial agent was below the detection limit of MIA. Presence of antimicrobial residues in the samples could inhibit metabolic activity and growth of sensitive bacteria so that the acidification of the medium does not occur. Hence, when the colour remained purple or the colour of the sample was clearly different to that of the negative control, the sample contained antimicrobial residues at a concentration above the detection limit of the assay. The samples with light shades of purple colour indicated the presence of antimicrobial residues near the detection limit of MIA in microplates and could be considered positive (Nagel et al., 2009; Kožárová et al., 2020). Photometric measurement results were expressed in relative absorbance according to the following equation; the limit of detection was assessed as the antimicrobial concentration that produces 45% of the maximum relative absorption (Althaus et al., 2003; Dang et al., 2011):

$$A=\left(A_{x}-A_{0}\right)/\left(A_{100}-A_{0}\right)$$

where *A* is the relative absorbance, *A_x_* is the absorbance of fluid sample with ‘*x*’ antimicrobial concentration, *A*_0_ is the absorbance of antimicrobial‐free fluid sample (negative control), and *A*_100_ is the absorbance of fluid sample with the antimicrobial concentration that produces 100% positive results (positive control).

### 2.5 Dose‐response curves

The dose‐response curves of the antimicrobial agents were constructed according to the Codex Alimentarius guidelines (Codex Alimentarius, 2016). Non‐linear regression of log concentrations vs. normalized responses of relative absorption was constructed by Graph Pad Prism 9.2.0 software.

### 2.6 Sensitivity and specificity study

The sensitivity is defined as the ratio between the number of positive samples obtained and the total number of true‐positive samples, whereas the specificity is defined as the ratio between the number of negative detected samples and the total number of true‐negative samples (Dang et al., 2010). These parameters, respectively, were determined by analysing of 100 samples including 50 spiked (at the level of MRL) and 50 blank chicken kidney tissue and expressed as percentage. Evaluation protocol was based on what described in Section 2.4. The results were interpretated visually by two trained persons as negative (pale to yellowish colour) and positive (shades of purple colour).

## 3 RESULTS

### 3.1 Detection capabilities

**FIGURE 2:**
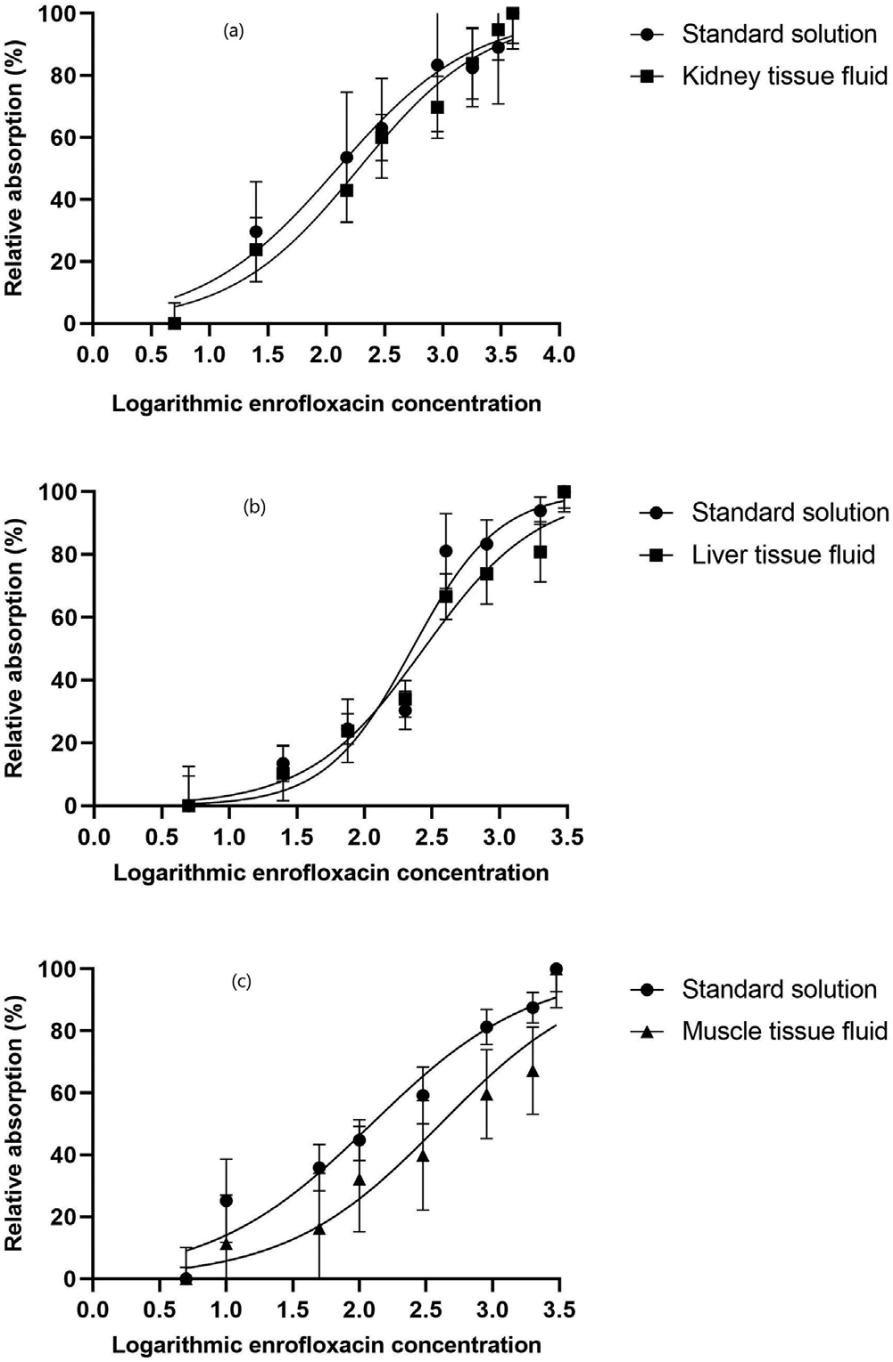
Dose‐response curves for the effect of enrofloxacin concentrations upon the relative absorption of microbial inhibition assay in chicken kidney (a), liver (b) and muscle (c). Standard solution: distilled water as antimicrobial agent diluent. Each point represents the mean of 11 determinations.

**FIGURE 3:**
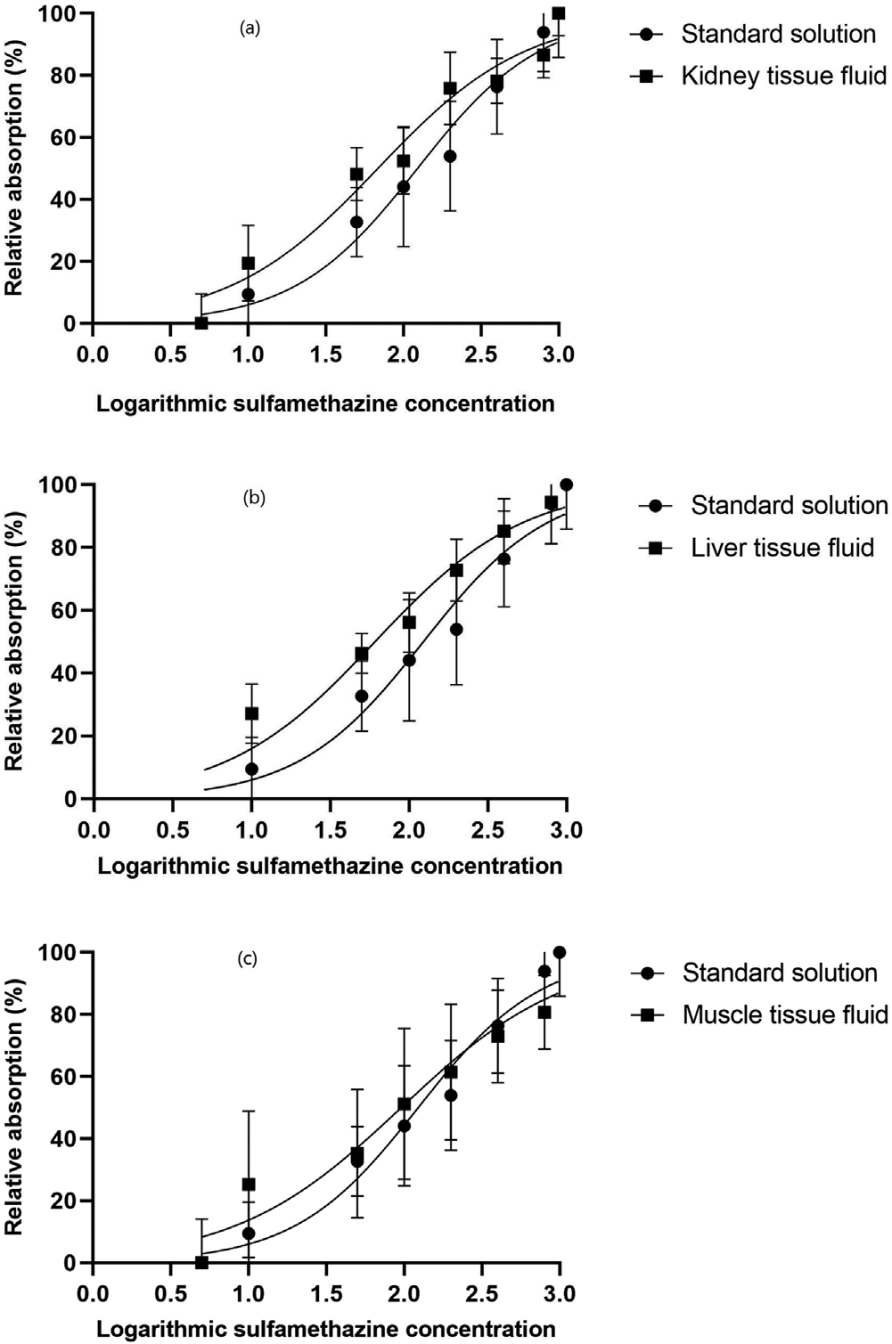
Dose‐response curves for the effect of sulfamethazine concentrations upon the relative absorption of microbial inhibition assay in chicken kidney (a), liver (b) and muscle (c). Standard solution: distilled water as antimicrobial agent diluent. Each point represents the mean of 11 determinations.

The effects of the concentrations of ENR and SMT in different matrixes on the relative absorption are shown in Figures 2 and 3a–c, respectively. In the dose‐response curves in the figures, each point represents the mean value of 11 replicates. It can be observed that the effects of antimicrobial concentrations on relative absorbance responses in non‐linear curves were stimulatory. The detection capabilities of MIA in microplates for ENR and SMT residues in chicken tissues and the parameters of non‐linear regression models are shown in Table 2. Slope hill coefficients obtained for ENR residues in chicken kidney and muscle samples were lower than those determined for SMT in chicken tissue samples (0.78, 0.75 and 1, respectively). As indicated in the Table 2, it was found that the detection limits of the MIA were less than MRL for ENR and SMT in chicken kidney, liver and muscle tissue fluids except for ENR in muscle tissue fluid which was obtained at a higher level but still close to MRL value (136.3 μg/kg compared to 100 μg/kg MRL). Furthermore, detection limits obtained by MIA in microplates for both antimicrobial agents in standard solution was below or slightly above the determined values for each tissue sample. It was observed that the coefficients of determination (*R* ^2^) were relatively high, from 0.7 for SMT in muscle to 0.92 for ENR in liver, indicating the appropriate outcome prediction capabilities of the regression model.

**TABLE 2:** The non‐linear regression model parameters and the detection limits of microbial inhibition assay in microplates for sulfamethazine and enrofloxacin residues in chicken tissues.

Antimicrobial agents	Samples	Coefficient of determination (*R* ^2^)	Degree of freedom	Hill slope	Detection limits (μg/kg)	EU‐MRL^a^ (μg/kg)
Sulfamethazine (SMT)	Kidney	0.89	87	1.00	23.21	100
	Liver	0.88	87	1.00	62.03	100
	Muscle	0.70	87	1.00	46.95	100
	Standard solution^b^	0.84	87	1.00	26.26	–
Enrofloxacin (ENR)	Kidney	0.90 (0.77)^c^	86 (86)	0.78 (0.74)	124.03 (110.40)	300
	Liver	0.92 (0.91)	87 (86)	1.00 (1.35)	90.02 (110.14)	200
	Muscle	0.76 (0.9)	86 (86)	0.75 (0.71)	136.38 (98.15)	100

^a^ European Union maximum residue levels.

^b^ Standard solution: distilled water as antimicrobial agent diluent.

^c^ Values associated with analysis of enrofloxacin in standard solution were presented in parentheses as different spiked levels were evaluated according to the MRL of enrofloxacin in tissues.

### 3.2 Sensitivity and specificity

The MIA in microplates had a sensitivity of 88% for ENR, with 44 samples being positive for ENR (true‐positive) and 6 being negative (false‐negative); nevertheless, higher sensitivity rate was obtained for SMT; in case, 46 samples being positive and 4 being negative, therefore, yielding a sensitivity of 92%. Results regarding specificity study showed that no false‐positive rate was found in the test.

## 4 DISCUSSION

There are several microbial screening tests developed to detect antimicrobial agents especially beta‐lactams in milk which are based on the inhibition of *G. stearothermophilus* but there is little research on microbial inhibition methods for chicken samples (Althaus et al., 2003; Le Breton et al., 2007; Molina et al., 2003). Many commercial microbial inhibition kits cannot suitably detect ENR as well as other quinolone antimicrobials in various matrixes because the indicator microorganism, *G. stearothermophilus*, in these tools is very insensitive to quinolones (Beltrán et al., 2015; Linage et al., 2007; Stead et al., 2004).

Most of the analytical methodologies for veterinary antimicrobial drugs has been developed and validated by using spiked tissue samples or even only standard antimicrobial solutions (Myllyniemi et al., 2000). The results in the present study showed that MIA using *B. licheniformis* as indicator could detect ENR and SMT residues in chicken spiked samples of kidney, liver and muscle. However, the capability in detection showed some variability in ENR as compared with SMT residues as lower values of slope hill coefficient were obtained for ENR in kidney and muscle tissue fluids and their respective diluents. Lower values of slope hill coefficient by applying regression model which was observed in ENR in kidney and muscle indicate a lesser increase in relative absorbance with the concentration and consequently decreased sensitivity in the detection capability of the method.

In the present study, moderate and small amounts of ENR and trimethoprim were used to enhance the sensitivity of the test to quinolones and sulfonamides, respectively. It has been reported that adding moderate concentrations of target antimicrobial agents to the culture media can improve detection capability of MIA (Tumini et al., 2019). Nevertheless, as the bacteriostatic mechanisms of selected sensitivity improvers including trimethoprim and ENR are different and could produce synergistic reaction, they might have positive effect on the detection capability of the test to other antimicrobials at the same time (Wu et al., 2019).

According to our results, detection limits were higher for ENR in kidney and muscle and SMT in liver and muscle tissue fluids than the values determined in antimicrobial agent diluent. This could be due to the matrix components in tissue fluids which were not present in antimicrobial diluent. However, the detection capabilities of the MIA for both antimicrobial agents in antimicrobial agent diluent were comparable to those determined in kidney, liver and muscle tissues and except for ENR in muscle were below the respective MRLs. Therefore, matrix components did not negatively affect the detection capabilities of the test. The detection limits of this study for ENR and SMT in chicken kidney, liver and muscle were lower than those reported by Nagel et al. (2013) for a bioassay in microplates by using of *G. stearothermophilus* (160 μg/L of ENR and 570 μg/L of SMT). Similarly, our results showed lower detection limits for evaluated antimicrobials as compared with Wu et al. (2019) who found detection limits of 400 μg/L for ENR, 300 μg/L for sulfadimidine and 150 μg/L for sulfadiazine in chicken egg samples by applying indicator microorganism *G. stearothermophilus*. Detection limit of SMT in the present study was similar to those reported for sulfonamides by Nouws et al. (1999) which was 40 μg/L of SMT, 30 μg/L of sulfadiazine and 20 μg/L of sulfadimethoxine for an assay in Petri dishes using *B. subtilis*. Tumini et al. (2019) evaluated MIA in microplates using *B. licheniformis* for the detection of quinolone and sulfonamide residues in milk. They detected ENR at 98 μg/L, sulfadiazine at 76 μg/L and sulfathiazole at 87 μg/L, lower than the respective MRLs. By comparing results of the present study and the results published by Tumini et al. (2017, 2019), it was revealed that obtained detection limits for ENR in chicken kidney and muscle was slightly higher (124.03 and 136.38 μg/kg, respectively) but the ENR in liver was at a similar level (90.02 μg/kg). Moreover, the detection limits for sulfonamide in our study were lower than those determined by Tumini et al. (2019) which could be related to differences in matrix types and composition of bioassays. Considering the above results, findings of this study indicated that MIA in microplates using *B. licheniformis* for ENR and SMT detection in chicken tissues could detect residues at the levels below or slightly above the respective MRLs compared to studies using *G. stearothermophilus* which mostly found higher detection limits for these antimicrobials. Moreover, the MIA in microplates yields results in a shorter detection time as compare with Petri dish methods.

It has been reported that matrix *pH* and the presence or absence of nutrients in matrixes may affect the detection time of microbiological inhibition tests by influencing on bacteriostasis developed by antimicrobial agents and the bacterial growth respectively (Wu et al., 2019). Chicken muscle tissue fluid was weakly acidic, whereas kidney and liver *pH* were in the neutral range. In agreement with previous reports, the detection time of the MIA in current study for muscle tissue fluids was evidently shorter than other analysed matrixes (3.25 h for muscle; 3.5 h for kidney; 3.66 h for liver and 3.5 h for antimicrobial agent diluent). Nonetheless, unlike previous reports, shorter detection time for tissue fluid matrixes compared to antimicrobial agent diluent was not observed in the present study which may be related to differences in sample preparation methods among different studies.

In general, the kidney as the major excretory organ of most drugs is known to have the highest antimicrobial residues and is considered a good sample for screening of antimicrobial residues in animal carcasses (Ezenduka et al., 2014). However, the indicator microorganisms used in microbiological assays are sensitive to the inhibitory activity of the natural bacteriostatic substances such as lysozymes present in the kidney which necessitates preheating treatment to diminish such substances before initiating the test (Wu et al., 2019). In the current study, the determination of the specificity in kidney tissue fluid showed that there were no false‐positive results in the test. In line with our results, high specificity rates of 98.1%, 99.5% and 98.9% in microbiological inhibition assays with indicator microorganisms *B. licheniformis, B. cereus, B. subtilis* and *Bacillus pumilus*, respectively, were reported in previous studies (Althaus et al., 2009; Nagel et al., 2013; Tumini et al., 2015). Further study by analysing samples of antimicrobial treated poultry by MIA of present study and chromatography‐based methods is needed to determine the correlation between screening and confirmatory tests results.

## 5 CONCLUSION

MIA in microplates could provide a method for the analysis of large number of samples in a short period of time (3–4 h). Moreover, in contrast to microbial inhibition tests in Petri dishes, which should be prepared just prior to use, microplates of this assay that contains sporulated bacteria could be stored at 4°C in the laboratory for upcoming use. Our results revealed that the assay using *B. licheniformis* as indicator microorganism could detect SMT and ENR residues at levels below or close to European Union‐MRL in chicken tissues including kidney, liver and muscle but offered lower capability for detection of ENR compared with SMT in kidney and muscle tissue samples.

## AUTHOR CONTRIBUTIONS

*Conceptualization; investigation; methodology; w*riting – original draft: Sara Mirzaie. *Investigation; methodology*: Fahimeh Jamiri. *Resources; writing – review and editing*: Majid Javanmard Dakheli. *Resources*: Saeed Mirdamadi.

## CONFLICT OF INTEREST STATEMENT

The authors had no conflicts of interest to declare.

## ETHICS STATEMENT

The authors confirm that the ethical policies of the journal, as noted on the journal's author guidelines page, have been adhered to and the appropriate ethical review committee approval has been received. The guidelines for animal use approved by the Animal Care Committee of the Iranian Council of Animal Care 1995 (No. IACUC95) were followed.

### PEER REVIEW

The peer review history for this article is available at https://www.webofscience.com/api/gateway/wos/peer‐review/10.1002/vms3.1293.

## Data Availability

The data that support the findings of this study are available from the corresponding author upon reasonable request.
